# Task demand mediates the interaction of spatial and temporal attention

**DOI:** 10.1038/s41598-024-58209-1

**Published:** 2024-04-22

**Authors:** Helena Palmieri, Marisa Carrasco

**Affiliations:** 1 Department of Psychology, New York University, New York, NY 10003 USA; 2 Center for Neural Science, New York University, New York, NY 10003 USA

**Keywords:** Neuroscience, Psychology

## Abstract

Psychophysical studies typically test attentional mechanisms in isolation, but in everyday life they interact to optimize human behavior. We investigated whether spatial and temporal attention interact in two orientation discrimination experiments that vary in task demand. We manipulated temporal and spatial attention separately and conjointly with well-established methods for testing each spatial or temporal attention. We assessed sensitivity (d′) and reaction time for every combination of spatial and timing cues, each of which was valid, neutral, or invalid. Spatial attention modulated sensitivity (d′) and speed (reaction time) across temporal attention conditions. Temporal attention modulated sensitivity and speed under high- but not low- task demands. Furthermore, spatial and temporal attention interacted for the high-demand task. This study reveals that task demand matters; in a simple task spatial attention suffices to improve performance, whereas in a more demanding task both spatial and temporal attention interact to boost performance, albeit in a subadditive fashion.

## Introduction

Spatial attention can be allocated covertly without concurrent eye movements. Endogenous (voluntary) spatial attention evokes stronger stimulus responses at pre-cued locations than uncued locations (review^1^). Spatial attention increases contrast sensitivity and spatial resolution at attended locations and decreases them at unattended locations^2–5^.

Attention is also allocated temporally. We can prioritize behaviorally relevant information at a particular moment in time^6–10^, for reviews see^11–13^. Temporal attention improves stimulus discriminability at foveal and parafoveal isoeccentric locations to a similar degree^14^. For both spatial and temporal attention, there are tradeoffs in processing. Benefits at the attended location have concurrent costs at unattended locations^1^. Likewise, perceptual improvements at the attended time have concurrent costs at earlier and later time points^7,8^.

Most psychophysical and neuroimaging studies investigate these two attentional mechanisms in isolation, but in everyday life they dynamically interact to optimize behavior. Coull and Nobre^15^ proposed a general neural system for allocating attentional resources and independent systems for modality, with preferential activation in the right parietal cortex for spatial attention, in the left parietal cortex for temporal attention, and bilateral activation when deploying both.

To characterize the relation between spatial and temporal attention, we need to determine whether they interact. Consider, for example, a circus juggler, precise timing and spatial accuracy is vital for her performance. Attending the apex of the trajectory (at a specific location and time) of each ball, does she get the sum of both attentional benefits? Or if the juggler is not attending to the ball location, does she still get the benefit of attending to the precise timing and successfully catch the ball?

These two attention systems could alter sensory representations dependently or independently. In principle, spatial and temporal attention effects could combine in three different ways, as: (a) the sum of the isolated effects, i.e. additive; (b) more than the sum of isolated effects, i.e. superadditive or (c) less than the sum of the isolated effects, i.e. subadditive. Here we investigate whether and how voluntary spatial and voluntary temporal attention interact with regard to both benefits and costs. In a previous study investigating how spatial and feature-based attention interact, the authors found additive effects with low-stimulus competition but superadditive effects with high-stimulus competition^16^. Thus, it is possible that whether we find additivity, superadditivity or subadditivity depends on the task demands.

Unfortunately, in the temporal literature, the terms “attention” and “expectation” have been used interchangeably and this oversight has permeated into the limited literature investigating the interaction between voluntary temporal and voluntary spatial attention (e.g., Refs.^10,15,17–24^). Here, we concentrate on the studies exploring the interaction of spatial and temporal attention. To do so, we follow the conceptual distinction between attention and expectation that has been established in the spatial and feature domains, in which the two factors have dissociable impacts on perception and neural responses^25–28^. Temporal attention is the prioritization of a task-relevant timepoint, and temporal expectation is the ability to predict the time of a particular stimulus regardless of task relevance^7,8,12,29–32^. In this study we manipulated temporal and spatial attention per se, rather than expectation, because the pre-cues indicated which locations and time points were most likely task-relevant, not most likely to contain particular stimuli.

We systematically investigated the effects of spatial and temporal attention independently and then measured their combined effect, all in the same task and with the same participants. Furthermore, we varied the task demands across two experiments, to explore whether this factor would modulate their possible deployed dependency and conjoined effect. We did so because previous research revealed that spatial and feature-based attention interact differently as a function of stimulus competition, which results in different task demand^16^.

We manipulated spatial and temporal attention to maximize their effects on visual performance, particularly on an orientation discrimination task. We varied temporal attention while equating expectation by using precues to direct voluntary temporal attention to specific stimuli in predictably timed sequences of brief visual targets^7,8,14,29,32^. Thus, this task requires temporally precise deployment of attention to a relevant time point that varies from trial to trial.

Our experimental design follows a previous study on the interaction of voluntary spatial and feature-based attention^16^. Our design enables us to isolate the effects of spatial and temporal attention and establish a baseline for comparison for the conjoint effect, and to assess both benefits of spatial and temporal attention at the attended location and/or time (brought about by valid cues) and costs at the unattended location and/or time (brought about by invalid cues) in comparison to neutral conditions. We investigated these effects of spatial and temporal attention, using sensitivity (dʹ) (primary dependent variable) as per Signal Detection Theory^33^, and response time (secondary dependent variable).

## Method

### Observers

Twelve observers participated in both experiments (aged 23–32 years, 6 females) for a total of 12 sessions and 6000 trials per observer. We based the sample size on a power analysis of the temporal attention effect, which we expected to be the most subtle effect. We derived the expected effect size (0.12) from previous investigations of voluntary temporal attention^8,14^ and involuntary temporal attention^30,31^, and evaluated the sample size needed for 80% power (using G*power: (F-test/ANOVA within factors measures), alpha error probability (0.05), number of groups (1)^34^. All observers provided written informed consent, had normal or corrected- to-normal vision and were neurotypical. All experimental procedures were in agreement with the Helsinki declaration and approved by the New York University Institutional Review Board.

### Stimulus

Stimuli were generated using a Linux (Ubuntu) adapted PC, with Psychophysics Toolbox Version 3 (PTB-3) and EyeLink toolboxes to control stimulus presentation and participant response collection. Stimuli were displayed on a 21″ Sony GDM-5402 CRT monitor with refresh rate of 85 Hz at a viewing distance of 57 cm. Observers’ heads were stabilized by a chin rest. A central black fixation cross subtended 0.5° of visual angle. Target stimuli were 4 cycles per degree sinusoidal Gabor gratings that subtended 3° of visual angle; one Gabor was presented in each quadrant of the display centered at 6° eccentricity. Targets were at 100% contrast for high visibility. The stimuli were presented on a medium gray background.

### Experiment One procedure

**Figure 1:**
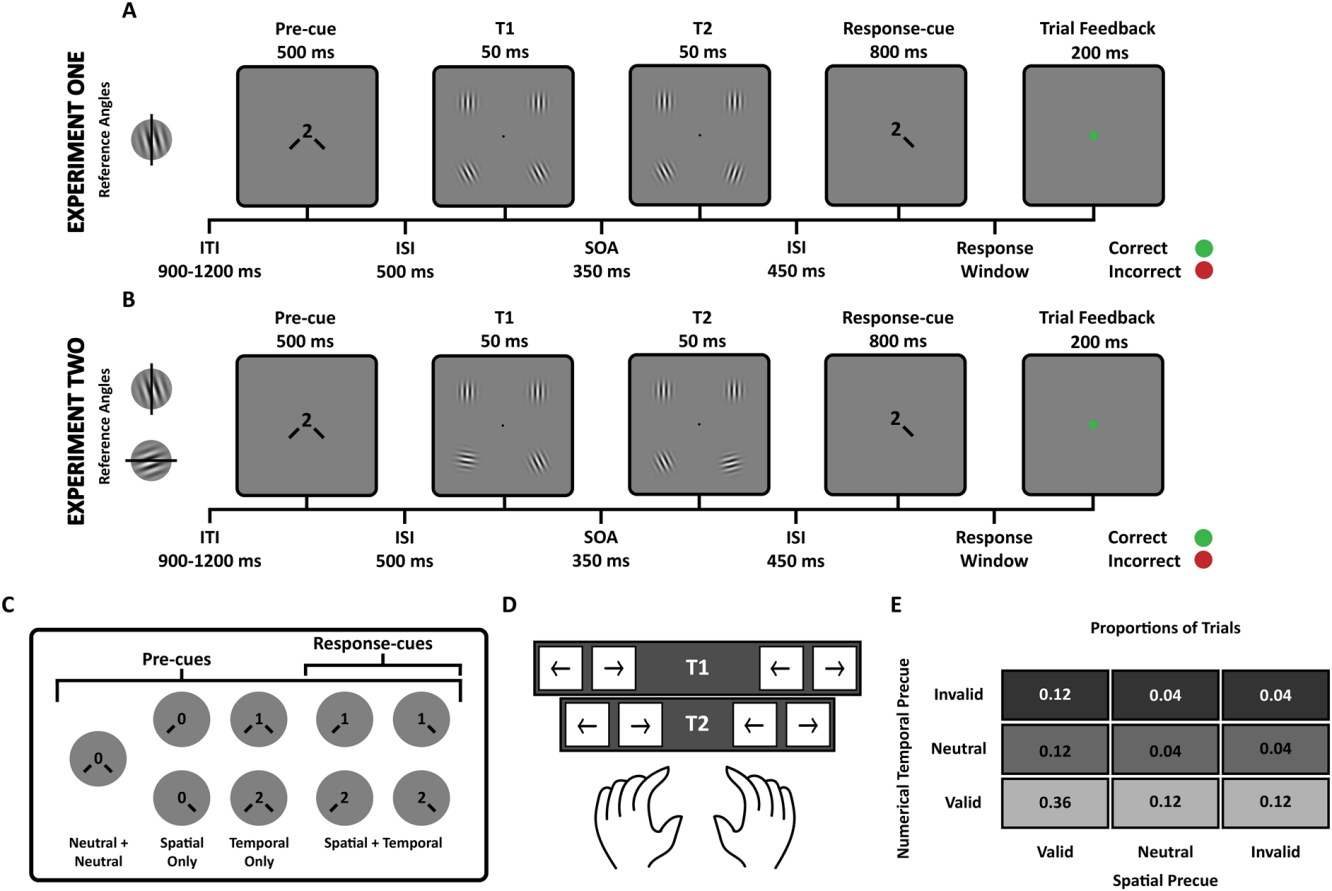
Experiment designs. (**A**) Example of a trial sequence in Experiment One with one reference angle. Participants judged the tilt (clockwise or counterclockwise) of a sinusoidal Gabor grating patch with respect to vertical. (**B**) Example of a trial sequence in Experiment Two with two reference angles. Participants judged the tilt (clockwise or counterclockwise) of a sinusoidal Gabor grating patch with respect to vertical and horizontal. Stimuli in both experiments and cueing conditions were presented in randomly interleaved trials. Tilt magnitudes in both experiments were determined per participant using a 3-up-1-down staircasing procedure. (**C**) Visualizing the types of pre-cues used to manipulate attention, shown at the beginning of a trial and the subset of those pre-cues are also used as response-cues. (**D**) Orientation report response key system. (**E**) Proportion of trials for each cue validity condition.

Observers were asked to discriminate the orientation of a tilted Gabor target grating (Fig. 1A). Each trial began with a jittered inter-trial interval (ITI) lasting between 900 and 1200 ms with only the black fixation point shown on the screen. Each trial had eight targets, four targets at Time One (T1) and four targets at Time Two (T2). For each presentation time (T1 and T2) there was one target in each visual field quadrant. Participants attended to only the two lower hemifield targets. The two targets in the upper hemifield were vertical. For Experiment One, targets were only tilted off of vertical (90°). Target presentation times (T1 and T2) were separated by a stimulus onset asynchrony (SOA) of 350 ms, which has been shown to enable benefits at the temporally attended time and costs at the unattended time^7^. Each Gabor in the lower hemifield was tilted slightly clockwise or counterclockwise (average tilt 3.93° [T1: 3.66°, T2: 4.20°], standard error 0.45° [T1: 0.41°, T2: 0.49°]) relative to vertical based on a tilt threshold estimated per participant during a pre-test thresholding session. The pre-cue (Fig. 1C) was shown for 500 ms and directed participants to allocate attention towards the relevant target (depending on the cue type, described below). The pre-cue presentation included both spatial and temporal information. The spatial pre-cue of either one or two lines, on either side of fixation, pointing towards one or both of the lower target locations, and the temporal pre-cue numbers centered at fixation indicated the timing (0 = no time information; 1 = T1; 2 = T2). To facilitate comparisons between the effects of spatial and temporal attention and assess their interaction, we utilized a visual cue that simultaneously directed observers to a location and time point. Together, all possible pre-cue feature combinations formed four categories:

1. Neutral + Neutral: left and right lines paired with a 0 at fixation, providing no information about time nor location.
2. Spatial Only (Time Neutral): a line pointing either to the left or right paired with a 0 at fixation, providing spatial information only.
3. Temporal Only (Location Neutral): lines pointing to the left and right paired with either a 1 or 2 at fixation, providing temporal information only.
4. Spatial + Temporal: a line pointing either to the left or right paired with either a 1 or 2, providing temporal and spatial information simultaneously. This subset of cues were also used as response-cues.

These endogenous cues have been used in multiple studies investigating spatial (e.g., Refs.^35–38^) and temporal^7,8,14,29,31,32,39^ attention, and are comparable in nature; both require a simple transformation—a single line indicates left or right and a single number indicates first or second. After the pre-cue offset, targets at T1 and T2 were presented for 50 ms, followed by an inter-stimulus interval (ISI) of 450 ms before the response-cue (Fig. 1C: spatial + temporal cues) appeared for 800 ms indicating to the participant which stimulus to report on. The combination of pre-cue and response-cue in the trial yielded a set of nine trial conditions (Fig. 1E). Per trial, there were equal chances of the target being in the T1 or T2 interval, in the left or right location and tilted off the horizontal or vertical axis. Following the response-cue offset (fixation point returned) participants gave their orientation report (clockwise or counterclockwise) for the stimulus indicated by the response-cue. Participants knew the cue was valid in most trials as the experimenter told them, “whereas the cue is not 100% valid it is valid the majority of the time, so please follow the directions of the cue”.

Participants used the key response system (Fig. 1D) to make their orientation (clockwise or counter-clockwise) reports. There were eight keys in total: the top row of keys corresponded to the targets presented at T1, the bottom set for the targets presented at T2, the left side of keys for the targets in the lower left and the right set of keys for the targets in the lower right. The motivation for having the key response system organized in this fashion (instead of having a simple two-key system: one key for counterclockwise and the second for clockwise) was to measure the types of swap errors people would commit in this task. For example, people may correctly report the orientation of the probed Gabor target but report the incorrect presentation time or incorrect location. After the report, participants received visual feedback for correct (green fixation point) and incorrect (red fixation point) responses on each trial based on the accuracy of the orientation report. If participants had the correct orientation report but reported the incorrect presentation time or location of the target, the incorrect dimension was prompted with text below the fixation point: “time” for timing mistakes and “space” for location mistakes. Additionally, participants received feedback regarding their performance (X percent correct) at the end of each block.

Observers completed approximately six 1-h sessions for the experiment across different days. Across the experiment participants completed 30 blocks of 100 trials (5 blocks per day and a short break was enforced after every block) with all combinations of cue types (Fig. 1E) in a randomized order for each block, resulting in 3000 trials collected from each participant across all sessions for Experiment One.

### Experiment Two procedure

The second experiment was conducted three months after the first one. In this experiment, the goal was to have a more demanding task, while stimulus discriminability was equated in the neutral-spatial and neutral-temporal condition via thresholding, so that spatial and temporal attention would have equal opportunity to benefit performance at the attended space and/or time and hamper performance at the unattended space and/or time. Piloting data revealed that once the trials were interleaved across the 9 different cueing conditions, sensitivity was a bit lower and reaction time was higher in Experiment Two (see next point), consistent with the increased task demand.

It was identical to the first, except that the two behaviorally relevant targets in the lower visual field were either tilted slightly relative to horizontal (0°) or vertical (90°), independently within each interval (they could be both off vertical, both off horizontal, or one off vertical and the other off horizontal, with a random location) and across intervals; i.e. the orientation of each stimulus is drawn randomly and independently from the others (Fig. 1B). Participants were informed that they should consider two cardinal reference orientations (randomly intermixed) as points from which the stimuli could be tilted. They were then asked to assess whether the tilt was clockwise or counterclockwise with respect to the relevant meridian (horizontal or vertical), which were intermixed across trials. We increased the task demand because the effects of spatial and feature-based attention on perception interact as a function of task demand^16^. Each Gabor in the lower hemifield was tilted slightly clockwise or counterclockwise (average tilt 5.29° [T1: 5.35°, T2: 5.22°], standard error 0.37° [T1: 0.40°, T2: 0.35°]) relative to vertical or horizontal based on a tilt threshold estimated per participant.

### Training & orientation thresholding

For both experiments, observers completed a practice block to familiarize themselves with the task and the response key system. Observers also completed a thresholding block with the neutral–neutral pre-cue (no spatial and no temporal information) to determine tilt thresholds to assure approximately 82% accuracy within a block. The tilt threshold per stimulus presentation time was estimated with a 3-up-1-down staircase^40^.

### Eye tracking

For both experiments, online eye tracking using the EyeLink 1000 Plus (SR Research Company) ensured central fixation throughout the trial. Secured fixation would initiate the trial, and fixation would be required before each cue and target presentation. If participants broke fixation (beyond 1.5° of visual angle) the trial would end and be repeated at the end of the block.

### Data analysis

To investigate the interaction between spatial and temporal attention we used discriminability (sensitivity) and reaction time. As in many previous studies, we operationalize and manipulate these constructs via spatial and temporal cues. Sensitivity (d′) was calculated for each target cueing condition. Correct reports of clockwise orientations in the lower left and right quadrant were arbitrarily coded as hits, and incorrect reports of counterclockwise orientations in the lower left and right quadrant were coded as false alarms. Additionally, we applied the log-linear rule^41^ to our d′ calculations to result in conservative estimations of sensitivity when infinite values of d′ occurred. To determine dependency an ANOVA was run. Specifically the interaction term’s significance will determine whether these systems were dependent (significant) or independent (not significant). We also calculated and compared d′ value ratios (spatial valid/spatial invalid and temporal valid/temporal invalid) to determine how these attentional mechanisms combined: superadditive, additive, or subadditive. Reaction time was our secondary measure, where we computed the average latency in each cueing condition between the response-cue onset and the key response. Moreover, we calculated the benefit/cost indices (for both sensitivity and reaction time) and the balanced integration score (BIS^42^) to combine the effects seen in sensitivity and reaction time. The BIS calculation gives equal weight to percent correct and reaction time performance measurements by converting percent correct and reaction time into z-scores, thereby bringing them to the same scale and subtracting the reaction time z-score from the percent correct z-score.

## Results

To compare the effects of spatial and temporal attention as well as their interaction as a function of task demand we conducted the following analyses. First, we assessed whether spatial or temporal attention or their interaction are similar or different as a function of task demands (Experiment One vs. Experiment Two) via a three way-ANOVA (2 experiments × 3 spatial cues × 3 temporal cues) on sensitivity. The only significant interaction was that of experiment x temporal cue, (*F*(2, 22) = 3.90, p = 0.036, *η*G2 = 0.26), experiment did not interact with spatial cue, (*F*(2, 22) < 1) or with spatial x temporal cues, (*F*(4, 44) < 1). All main effects were significant: experiment *F*(1, 11) = 18.19, p = 0.0013, *η*G2 = 0.62, spatial cue *F*(2, 22) = 124.70, p < 0.001, *η*G2 = 0.92, and temporal cue *F*(2, 22) = 14.85, p < 0.001, *η*G2 = 0.57. Overall discriminability was higher for Experiment One than Two (1.60 v. 1.34). Sensitivity was higher for the valid spatial cue than for the neutral, which in turn was higher than for the invalid cue. Likewise, sensitivity was higher for the valid temporal cue than for the neutral, which in turn was higher than for the invalid temporal cue. Thus, this test revealed that the effects of the temporal cue on sensitivity varied as a function of task demands: the higher the demand, the greater the temporal attention effects.

We also performed the equivalent three way-ANOVA on reaction time, our secondary measurement. There were main effects of spatial cue, (*F*(2, 22) = 44.99, p < 0.001, *η*G2 = 0.80) and temporal cue, (*F*(2, 22) = 40.12, p < 0.001, *η*G2 = 0.78), but not of experiment (*F*(1, 11) < 1). Reaction time was faster for the valid spatial cue than for the neutral, which in turn was faster than for the invalid cue. Likewise, reaction time was faster for the valid temporal cue than for the neutral, which in turn was faster than for the invalid temporal cue. The effects of temporal attention were more pronounced for the valid spatial than the neutral and invalid conditions, and the effects of spatial attention were more pronounced for the valid temporal than the neutral, which in turn was more pronounced than for the invalid conditions. Hence, spatial and temporal cues modulated reaction times, always in agreement with the effects of sensitivity, indicating there was no speed-accuracy trade-off.

### Experiment One

**Figure 2:**
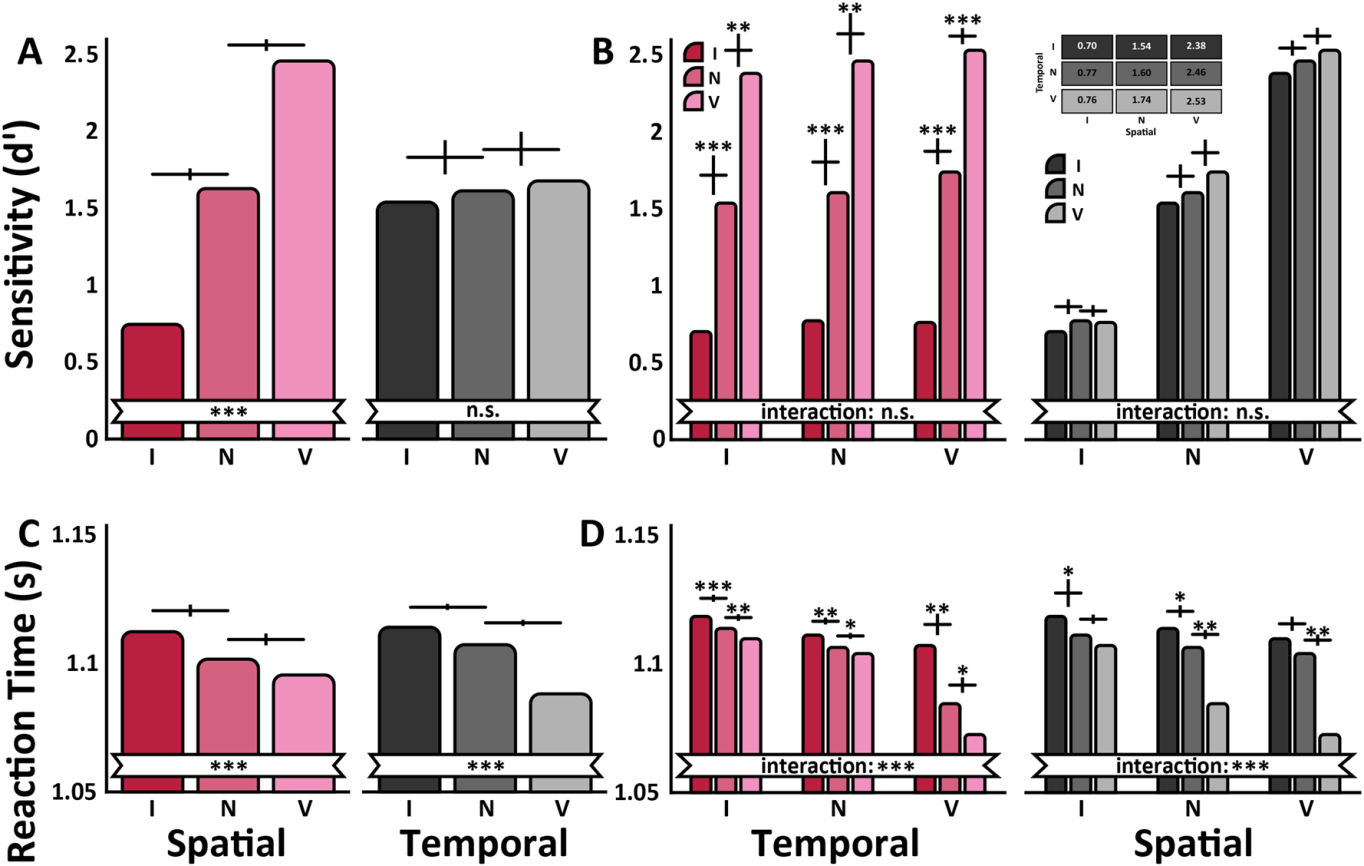
Experiment One results. (**A**, **B**) depict the same dʹ data plotted in different configurations to highlight specific effects. (**A**) Isolated Spatial Attention dʹ Values: Spatial attention effect collapsed across temporal attention conditions. Isolated Temporal Attention dʹ Values: Temporal attention effect collapsed across spatial attention conditions. (**B**) Averaged dʹ Values: Temporal pre-cue validity on the x-axis (Invalid, Neutral and Valid). Spatial pre-cue validity represented with colored bars: Invalid (red), Neutral (mauve), Valid (pink). Averaged dʹ Values: Spatial pre-cue validity on the x-axis (Invalid. Neutral and Valid). Temporal pre-cue validity is represented with colored bars: Invalid (black), Neutral (medium-grey), Valid (light-grey). Inside this panel is a table showing the mean dʹ value numerically for each cue validity condition. (**C**, **D**) depict the same RT data plotted in different configurations to highlight specific effects. Temporal pre-cue validity is represented with colored bars: Invalid (black), Neutral (medium-grey), Valid (light-grey). For all graphs, error bars indicate ± one standard error of the differences between the bars, ***p < 0.001, **p < 0.01, *p < 0.05.

A three-way ANOVA (3 spatial cues × 3 temporal cues × 2 time interval) on sensitivity (dʹ) revealed a main effect of spatial cue, F(2,22) = 33.28, p < 0.001, ηG2 = 0.75 (Fig. 2A**:** left panel), and no significant effect of temporal cue, F(2,22) = 2.83, p = 0.08, ηG2 = 0.21 (Fig. 2B**:** left panel). The only significant interaction was between the time interval and the spatial cue, F(2,22) = 3.46, p = 0.049, ηG2 = 0.24; the spatial cue was more pronounced in T1, (F(2,22) = 36.24, p < 0.001, ηG2 = 0.78, than T2, F(2,22) = 27.07, p < 0.001, ηG2 = 0.71. There was no interaction between the two cue types, *F*(4, 44) < 1 (Fig. 2B). Within each temporal cueing condition, spatial sensitivity (d′) was better in the valid condition, and worse in the invalid than in the neutral condition (Fig. 2B: left panel).

**Figure 3:**
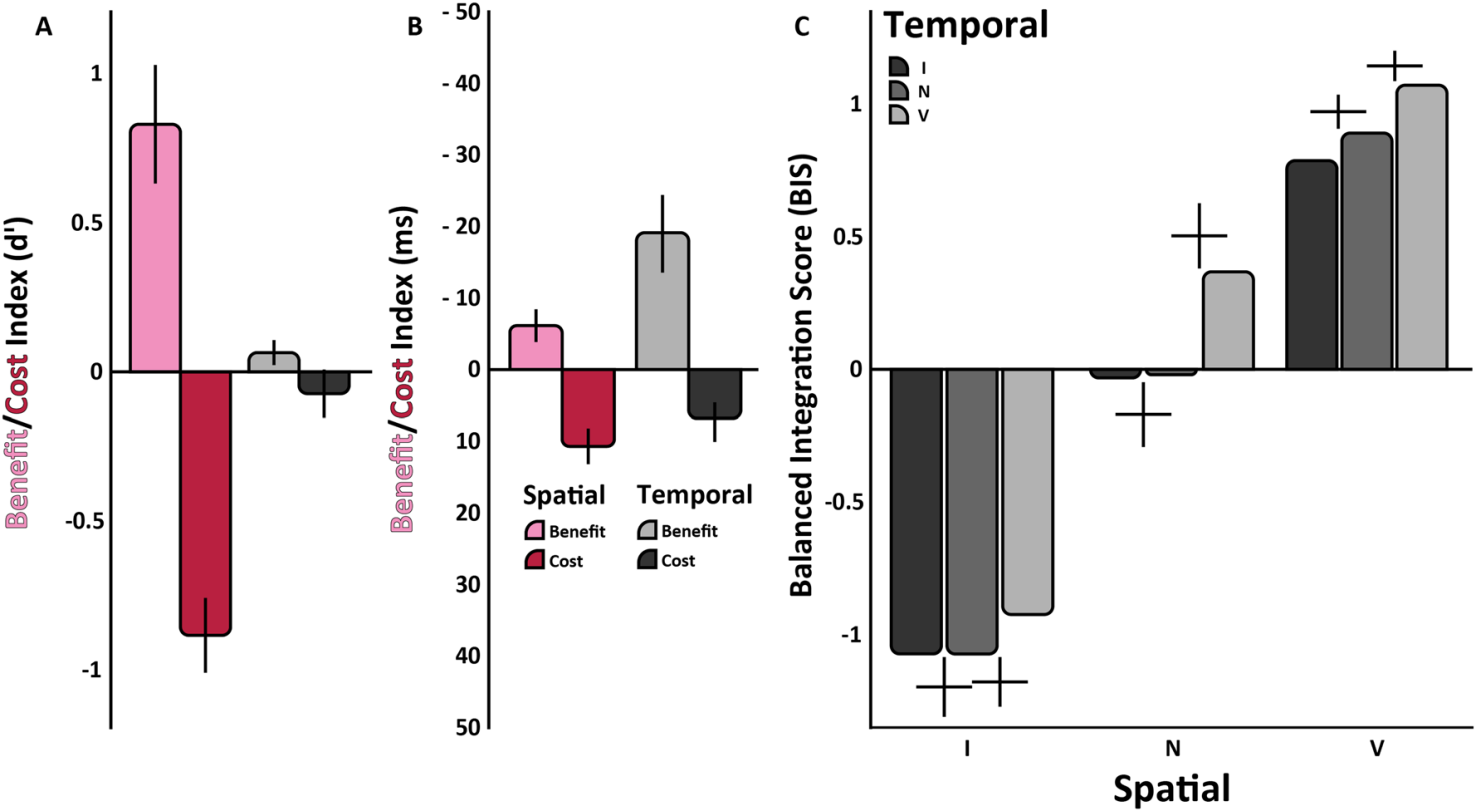
Experiment One benefit/cost indices and BIS values. (**A**) Benefit/Cost Index for Sensitivity (dʹ): Spatial valid pre-cue benefit (pink) and spatial invalid pre-cue cost (red). Temporal valid pre-cue benefit (light-gray) and temporal invalid pre-cue cost (black). (**B**) Benefit/Cost Index for Sensitivity (RT). (**C**) BIS for Experiment One: Spatial pre-cue validity on the x-axis (Valid, Neutral and Invalid). Temporal pre-cue validity is represented with colored bars: Invalid (black), Neutral (medium-gray), Valid (light-gray). A value of 0 conveys a lack of influence on performance; positive values denote enhanced performance; negative values denote diminished performance. For all graphs, error bars indicate ± one standard error of the mean.

We also examined reaction time (Fig. 2C,D) from the onset of the response-cue until the keyboard response. The analysis indicates that there were no speed-accuracy tradeoffs. The three-way ANOVA revealed main effects of spatial cue, F(2,22) = 13.90, p < 0.001, ηG2 = 0.56, and temporal cue, F(2,22) = 9.71, p < 0.001, ηG2 = 0.469 (Fig. 2C). The only interaction was between the spatial and temporal cue, F(4,44) = 5.87, p = 0.007 (ηG2 = 0.39) (Fig. 2D). Overall, the effects of spatial attention for sensitivity and speed were more pronounced than those of temporal attention, as illustrated by the benefit (valid-neutral) and cost (invalid-neutral) indices (Fig. 3A,B), except in the reaction time benefit, in which the opposite was the case (Fig. 3B). Specific values on which these calculations are based are presented in Fig. 2B, right panel.

Although there was no interaction between the spatial and temporal cues in sensitivity (d′) there was interaction between them in RT. To further assess the performance effect of each cue as well as their interaction, we calculated the balanced integration score (BIS) (Fig. 3C), which combines both sensitivity and speed. A two-way ANOVA on the BIS values revealed main effects of the spatial cue, F(2, 22) = 37.36, p < 0.001, ηG2 = 0.77 (Fig. 3C), and of the temporal cue F(2, 22) = 4.68, p = 0.02, ηG2 = 0.30 (Fig. 3C), but no interaction between them F(4, 44) < 1 (Fig. 3C). Thus, even when the effect of temporal cue was significant, it did not interact with spatial attention. BIS effects were the same for T1 and T2 (all interactions p > 0.1).

### Experiment Two

**Figure 4:**
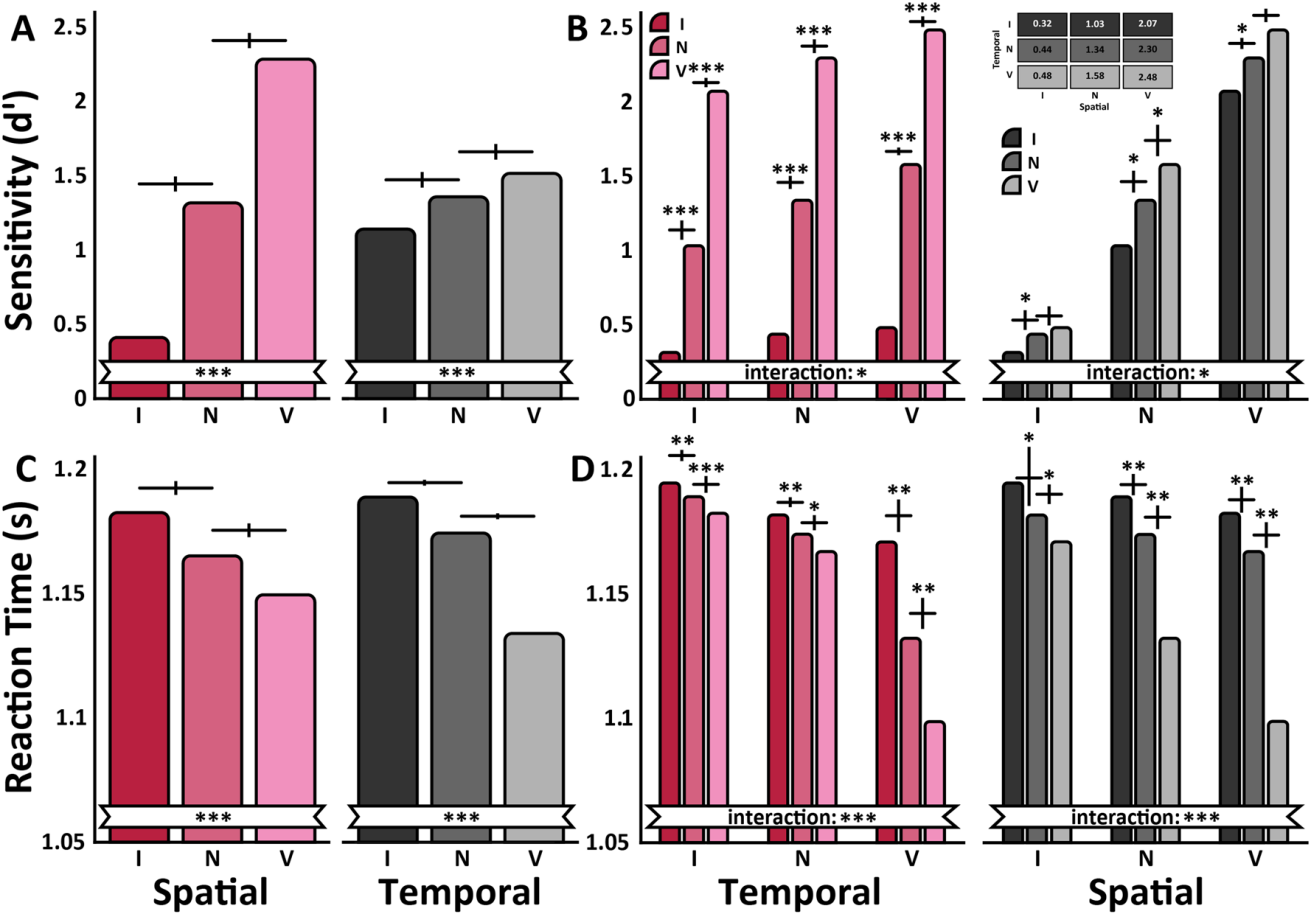
Experiment Two results. (**A**, **B**) depict the same dʹ data plotted in different configurations to highlight specific effects. (**A**) Isolated Spatial Attention dʹ Values: Spatial attention effect collapsed across temporal attention conditions. Isolated Temporal Attention dʹ Values: Temporal attention effect collapsed across spatial attention conditions. (**B**) Averaged dʹ Values: Temporal pre-cue validity on the x-axis (Invalid. Neutral and Valid). Spatial pre-cue validity represented with colored bars: Invalid (red), Neutral (mauve), Valid (pink). Averaged dʹ Values: Spatial pre-cue validity on the x-axis (Invalid. Neutral and Valid). Temporal pre-cue validity is represented with colored bars: Invalid (black), Neutral (medium-grey), Valid (light-grey). Inside this panel is a table showing the mean dʹ value numerically for each cue validity condition. (**C**, **D**) depict the same RT data plotted in different configurations to highlight specific effects. Temporal pre-cue validity is represented with colored bars: Invalid (black), Neutral (medium-grey), Valid (light-grey). For all graphs, error bars indicate ± one standard error of the differences between the bars, *p < 0.05, ***p < 0.001.

We conducted the same analyses as in Experiment One. A three-way ANOVA on sensitivity (dʹ) revealed main effects of spatial cue (Fig. 4A: left panel), F(2,22) = 122.23, p < 0.001, ηG2 = 0.918) temporal cues (Fig. 4A: right panel), F(2,22) = 20.80, p < 0.001, ηG2 = 0.65.

The 3-way interaction was not significant, but all the two-way interactions were significant. The significant interactions of time interval x spatial cue, F(2,22) = 4.09, p = 0.031, ηG2 = 0.27, and time interval x temporal cue, F(2,22) = 4.34, p = 0.026, ηG2 = 0.28, resulted from more pronounced effects of spatial cues for T2, F(2,22) = 113.95, p < 0.001, ηG2 = 0.91, than T1, F(2,22) = 72.83, p < 0.001, ηG2 = 0.87, as well as of temporal cues for T2, F(2,22) = 15.18, p < 0.001, ηG2 = 0.58, than T1, F(2,22) = 10.21, p = 0.0007, ηG2 = 0.48.

There was also an interaction of spatial × temporal cues F(4,44) = 2.64, p = 0.046, ηG2 = 0.19. There was a spatial cueing effect within each temporal cueing condition (Fig. 4B: left panel): sensitivity (d′) was better in the valid than neutral conditions, and worse in the invalid than neutral conditions for each temporal cue (all p < 0.001). The temporal cue effect manifested in greater sensitivity for the valid than neutral cue in the valid spatial (trend: p = 0.068) and neutral spatial (p = 0.02) but not in the invalid spatial (p > 0.1), and greater sensitivity for the neutral than invalid cue for all spatial cues (valid: p = 0.02; neutral: p = 0.034 and invalid: p = 0.039) (Fig. 4B**:** right panel).

We also examined reaction time (Fig. 4C,D). A three-way ANOVA on reaction time revealed significant main effects of spatial cue, F(2,22) = 18.82, p < 0.001, ηG2 = 0.63, and temporal cue, F(2,22) = 15.40, p < 0.001, ηG2 = 0.58. The three-way interaction, F(4,44) = 3.54, p = 0.014, ηG2 = 0.24, emerged because the effects of spatial cue, temporal cue and their interaction was more pronounced in T2 than T1 (T2: spatial, F(2,22) = 19.68, p < 0.001, ηG2 = 0.64; temporal, F(2,22) = 6.94, p = 0.0046, ηG2 = 0.39; spatial × temporal, F(4,44) = 4.65, p = 0.0032, ηG2 = 0.30; T1: spatial, F(2,22) = 10.38, p < 0.001, ηG2 = 0.49; temporal, F(2,22) = 6.56, p = 0.0058, ηG2 = 0.37; spatial × temporal, F(4,44) = 7.13, p < 0.001, ηG2 = 0.39). In short, reaction times mirrored the sensitivity data and showed that there were no speed-accuracy tradeoffs. Specific values on which these calculations are based are presented in Fig. 4B**,** right panel.

**Figure 5:**
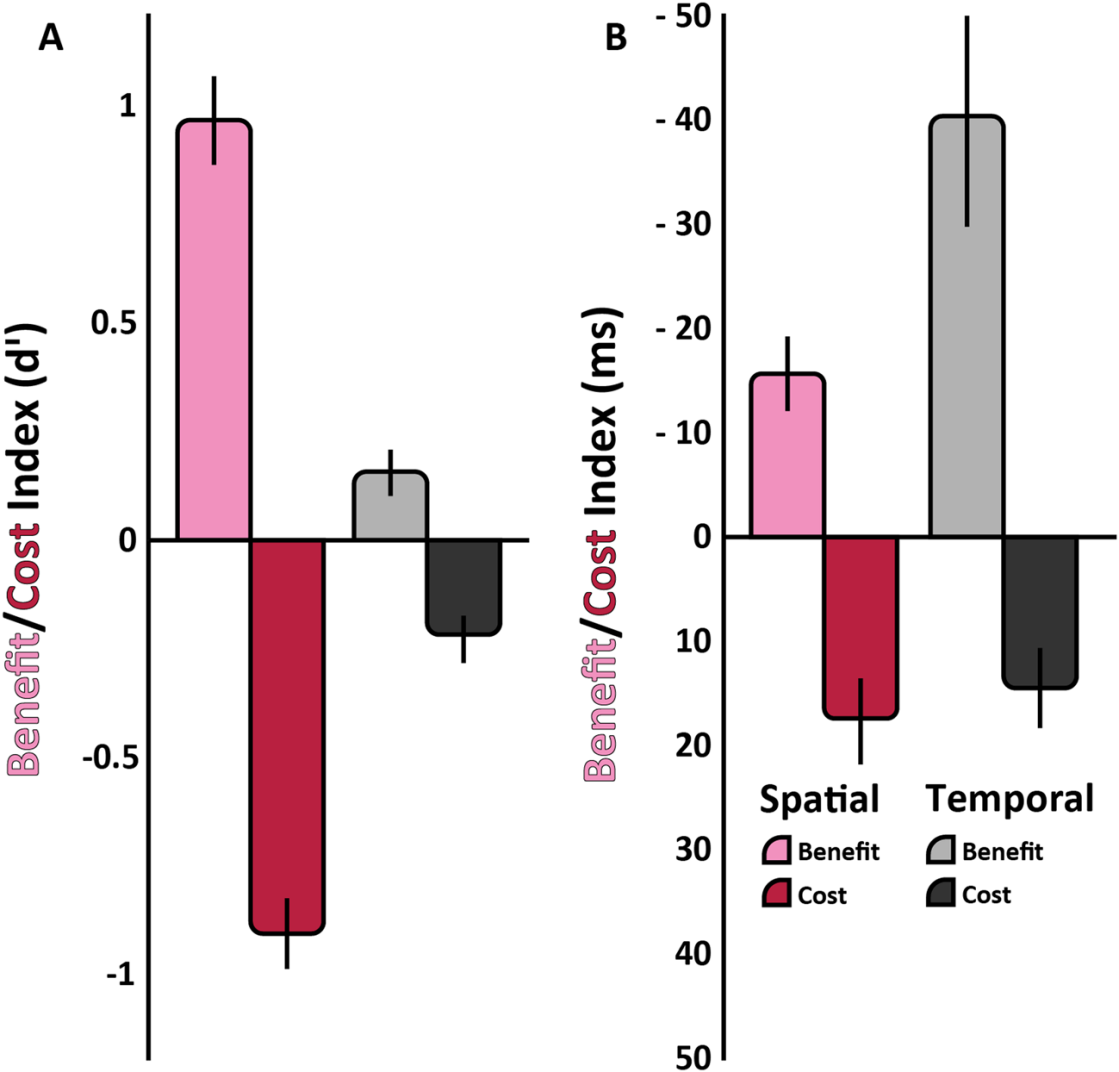
Experiment Two benefit/cost indices. (**A**) Benefit/Cost Index for Sensitivity (dʹ): Spatial valid pre-cue benefit (pink) and spatial invalid pre-cue cost (red). Temporal valid pre-cue benefit (light-gray) and temporal invalid pre-cue cost (black). (**B**) Benefit/Cost Index for Sensitivity (RT). For both graphs, error bars indicate ± one standard error of the mean.

Overall, the effects of the spatial cue for sensitivity and speed were more pronounced than those of the temporal cue, as illustrated by the benefit (valid-neutral) and cost (invalid-neutral) indices (Fig. 5A,B), except in the reaction time benefit, in which the opposite was the case (Fig. 5B).

The interaction of spatial and temporal cues was subadditive: the overall effect (valid–invalid) when attending to both the location and the timing of a stimulus was less than the sum of each individually. As can be seen in Fig. 4B, the spatial cue benefit on d′ was 1.86 when the temporal cue was neutral (left panel middle bar cluster: light pink bar [2.30]—red bar [0.44]) and the temporal benefit was 0.55 when the spatial cue was neutral (right panel middle bar cluster: light grey bar [1.58]—black bar [1.03]). When combined (temporal valid, left panel right bar cluster: light pink bar [2.48]—red bar [0.48]), the effect was 2.00, which is lower than the sum of the individual spatial and temporal effects (1.86 + 0.55 = 2.41). The interaction could also be described as subtractive: spatial attention subtracts d′ by a fixed factor d′ from the temporal attention modulation (same analysis as done in^16^). The overall subtractive factor can be approximated by examining the spatial valid/spatial invalid d′ ratios, which was higher for the temporal valid than the temporal invalid condition (12.33 [2.48/0.48] vs. 2.24 [2.07/0.36]), although the difference was not statistically reliable t(11) = 0.19. Alternatively, temporal attention subtracts d′ by a fixed factor d′ from the spatial attention modulation. The temporal valid/temporal invalid ratio was similar for the spatial valid and spatial invalid conditions (1.22 [2.48/2.07] vs. 1.12 [0.48/0.32]: t(11) = 0.89). Moreover, the study revealed that the temporal cue can enhance performance in cases of neutral spatial conditions, which had previously only been speculated about, and even in situations of spatial misdirection. In sum, the advantage of attending to both the stimulus’ location and timing was smaller than the combined advantage of focusing on each aspect separately.

## Discussion

We investigated whether and how endogenous spatial and temporal attention interact in a discrimination task and if so whether they have an additive, superadditive or subadditive effect.

There were significant effects of spatial attention and of temporal attention in both experiments, as manifested in measures of sensitivity, speed, and/or balanced integration score. In Experiment One, the effects of spatial attention were significant in both intervals but they were more pronounced in the first (T1) than the second (T2) interval. In Experiment Two, the effects of spatial and temporal attention were significant in both intervals, but for both sensitivity and speed, they were more pronounced in T2 than T1. Overall, the benefit and cost of spatial attention at the attended and unattended locations was more pronounced than the corresponding effects of temporal attention at attended and unattended times.

This study revealed that spatial attention has a pronounced effect across temporal attention conditions regardless of task demand (Figs. 2 & 4) both in sensitivity and speed, whereas the temporal attention effects were dependent on the demands in the task. Under higher task demands, temporal attention effects emerged in sensitivity and, to a greater extent in speed. Interestingly, the interaction of spatial and temporal attention on sensitivity (dʹ) was subadditive: the total benefit (d′) when attending to both the location and the timing of a stimulus was less than the sum of each individually. Similarly, the joint effects of spatial and feature-based attention systems interact with task demands, but this interaction is of a different nature: when stimulus competition was low spatial and temporal attention had an additive effect; when stimulus-competition was high they had a superadditive effect^16^. Thus, whereas the effects of spatial and feature-based attention potentiate each other, the effects of spatial and temporal attention can temper each other.

In a study using a blocked rapid serial visual presentation (RSVP), the authors concluded that combined attentional effects were independent and additive^21^. Although they measured the baseline isolated attention effects of spatial and temporal attention, their design was not balanced regarding the demands in both modalities (i.e., temporal cues reduced uncertainty more than spatial cues, as there were 15 frames and 4 locations), and their conclusion regarding additivity was based on reaction time analyses only. Interpretation of reaction time measures is limited as they reflect speed of processing, sensitivity and motor preparation (e.g. Refs.^8,43,44^).

Temporal attention can benefit performance when spatial attention is distributed across locations and even when misdirected. Temporal attention studies documenting both benefits and costs have exclusively used blocked spatial locations^7,8,14,29,32^. For the first time, we assessed the effects of spatial attention and found that the temporal attention effects in the valid spatial cueing condition in Experiment Two (Fig. 4B) were significant in both sensitivity and speed. In addition, the benefits and costs of temporal attention also emerged in the neutral spatial condition, which had not been tested before and only speculated over^8^, and there was an overall effect of temporal attention even when there was a spatial misdirection (i.e. with an invalid spatial cue, a valid temporal cue improved sensitivity compared to an invalid temporal cue, Fig. 4B right panel).

This is the first study that has found an interaction between spatial and temporal attention in a discrimination task and the first that has found temporal cue benefits even when spatial attention is distributed (spatial neutral) (Fig. 4B: right panel). Additionally, we analyzed the data by target type (tilted off-vertical and tilted off-horizontal) and found that the effects of spatial and temporal attention on sensitivity and reaction time performance were similar in both cases. The performance benefits and costs for both spatial and temporal attention and these effects interacted in Experiment One (only in RT) and in Experiment Two (in sensitivity and reaction time): the spatial attention effects were more pronounced for the valid temporal cue than the invalid temporal cue, and vice versa, the temporal attention effects were more pronounced for the valid spatial cue than the invalid spatial cue.

Similar reaction time findings have been reported in studies dealing with visual detection tasks^15,17,18,20,24^. The one study dealing with a discrimination task manipulated both temporal attention and expectation together and found a significant effect of temporal orienting at the attended location but not at the unattended location^10^.

The temporal attention task we used here, either with one (vertical) or two reference orientations (vertical and horizontal) has a constant visual working memory load for all trials (neutral, valid and invalid); moreover, in principle, both the stimuli on the first (T1) and second (T2) intervals could interfere with the processing of each other via either proactive and retroactive interference, respectively. This temporal attention task has been used in many studies (e.g.,^7,8,14,29,32,39,45^), and usually the effect of attention has been more significant for T1 than T2. In the present study, in Experiment One the effects of spatial attention were more pronounced in T2 than T1 whereas in Experiment Two, the effects of both spatial and temporal attention were more pronounced in T2 than T1. We think this difference is unlikely to be related to different working memory load, because in both experiments people had to hold the same information in visual working memory—four items with binding of location and identity information—and the same response alternatives: with one key-press, observers indicated the time, location and tilt of the target.

The benefit/cost indices show more pronounced effects of spatial attention than temporal attention, except that reaction time benefits were higher for temporal than spatial conditions, and these patterns were more prominent with higher task demands; i.e., they were more pronounced in Experiment Two (Fig. 5A,B) than Experiment One (Fig. 3A,B). In Experiment One, reaction time effects motivated the combined performance metric (BIS;^42^), which revealed that under low task demands (Fig. 3C) the temporal attention effects occurred most clearly when people are oriented correctly in space (spatial valid condition). These findings could be due to either being easier for observers to attend to a location rather than a timepoint or to observers assigning more weight to the spatial cues, and once they are misdirected in space, neutral or invalid information about time no longer impacts performance.

A recent review focused on the investigation of our capacity to consciously direct our attention to specific spatial locations and time intervals^46^. Although this review unfortunately overlooked the distinction between temporal expectation and temporal attention, it advances our understanding on the coordination of these attentional mechanisms. It highlights the benefits of using discrimination rather than detection tasks and of manipulating task demand for spatial and temporal attention within the same participants. Fortunately, this is exactly what we did: we manipulated endogenous spatial and temporal attention task demands within the same individuals using the same basic orientation discrimination task.

In this study, we utilized a discrimination task that involved using symbolic cues. Typically, distinct auditory tones have been used to manipulate temporal attention (T1—high tone and T2—low tone;^7,8,14,29,32^ (Duyar et al. 2024)). Here, to facilitate comparison between the effects of spatial and temporal attention, we employed a visual cue that simultaneously instructed observers how to deploy both types of attention. Our task had two different levels of perceptual demands. Similar to the study conducted by Rohenkohl et al.^10^, we identified interactive effects in the situation where perceptual demands were high. To enhance the reliability of these findings, we took steps to ensure that our task first elicited distinct effects related to temporal and spatial attention independently, before assessing how these effects combined. Our study furthers existing research by incorporating two distinct levels of perceptual demands, using the same simple symbolic cues, within the same set of participants across both experiments. Hence, we have the ability to make comparisons between experiments and conditions. Moreover, in the temporal attention literature, there has been ambiguity regarding whether temporal attention operates in a manner tied to spatial aspects^10,12,18^ or independently from them^21,24,47^. For the very first time, our findings clarify that temporal attention is not confined to spatial specificity. Notably, we observed temporal attention benefits and costs even when participants were directed to allocate their spatial attention across two different locations.

Our experimental design was set up to maximize the effects of both spatial and temporal attention, uses both performance and reaction time, and utilizes known protocols that elicit isolated and combinatory attention effects, thus enabling us to systematically investigate their interaction. In summary, our findings suggest that when task demands are low, spatial attention significantly affects performance, while temporal attention has little impact, and these mechanisms do not interact. However, as task demands increase, both spatial and temporal attention play a role and work together to enhance performance, albeit in a subadditive fashion. In essence, the more demanding the task, the greater the interaction between these attention systems, leading to improved performance.

## Data Availability

The dataset generated during and/or analyzed during the current study is available upon discretion from the corresponding author, Helena Palmieri.
